# Editorial: Emerging Mechanisms in Neuronal Signaling: From Cell Biology to Pathogenesis

**DOI:** 10.3389/fncel.2020.00257

**Published:** 2020-08-31

**Authors:** Mario Eduardo Guido, Gabriela Alejandra Salvador, Alejandra del Carmen Alonso

**Affiliations:** ^1^Departamento de Quimica Biologica “Ranwel Caputto”, Facultad de Ciencias Quimicas, CONICET Center for Research in Biological Chemistry, National University of Cordoba (CIQUIBIC), Cordoba, Argentina; ^2^Departamento de Biología, Bioquímica y Farmacia, CONICET- Instituto de Investigaciones Bioquímicas de Bahía Blanca, Universidad Nacional del Sur, Bahía Blanca, Argentina; ^3^Department of Biology and Center for Developmental Neuroscience, College of Staten Island, Staten Island, NY, United States; ^4^Biology Program, Graduate Center, The City University of New York, New York, NY, United States

**Keywords:** lipid signaling, neurodegeneration, retina, metabolism, cell signaling, oxidative stress, transcription factors, developmental disorders

Unraveling the molecular processes involved in the genesis, differentiation, and cell death of the nervous system is an intense and continual interest of the neuroscience community. In recent years, the preponderance of research focused upon signal transduction mechanisms relying on protein cascades, but more information is needed on the role and function of other molecular mechanisms. These molecular mechanisms include but not limited to: lipid mediators (sphingolipids, fatty acids, glycerophospholipids, etc.), lipid-binding proteins (ApoD, PPAR, etc.), protein-lipid interactions (c-Fos-lipid synthesizing enzymes), protein misfolding and not fully characterized membrane-protein receptors. Signal transduction events triggered by bioactive lipids and related transcription factors (immediate early genes, metabolic regulators, etc.) now receive special attention as an important nodal regulatory process. Deregulation of lipid-mediated processes is also linked to neurodegenerative diseases [Parkinson (PD), Alzheimer (AD), and retinopathies] and proliferative disorders (brain cancer and diabetic retinopathy). Moreover, the modern lifestyle (hypercaloric diets, continuous artificial light exposure, sedentary life, aging, stress) impacts on lipid signaling and metabolism, and can alter brain function, physiology, and behavior. Focused on this broad spectrum of underlying mechanisms related to molecular and cellular neuroscience, a Research Topic call elicited expansive, research interest and submissions from among international laboratories. As a result of this interest, 33 contributions are accepted and published on the molecular mechanisms as described above. One hundred and fifty-eight authors from research laboratories located in nine countries: North and South America (Argentina, Canada, Chile, and USA), Asia (China), and Europe (France, Poland, Spain, Switzerland), contributed to the accepted, peer-reviewed articles.

## Glia-neuron Crosstalk From Biology to Pathophysiology

Interesting contributions to this Research Topic includes the characterizations of the glia-neuron communication and interaction that is reported by Pascua-Maestro et al. and Volonté et al. and reviewed by Barber and Raben. Particularly, Pascua-Maestro et al. clearly demonstrated that glial cells rescue neurons exposed to cellular stress by astrocyte-secreted extracellular vesicles loaded with the lipid binding protein: ApoD. These observations highlighted the neuro-protective role of ApoD to promote neuronal survival during oxidative stress. In the future, novel therapeutics may involve ApoD-loaded exosomes because these exosomes can cross the blood-brain barrier to treat neurodegenerative diseases. Astroglial cells secrete lipid signals that can modulate functionally both presynaptic and postsynaptic neurotransmissions and consequently brain activity. The lipid compositions of the pre- and post-synaptic membranes of neurons impact functions involving vesicle fusion and receptor mobility, and strongly suggest an essential lipid-mediated communication between glia and neurons. Nevertheless, lipid metabolism controlling the interactions among different cell lineages is a new frontier in neuroscience. Barber and Raben reviewed the published research on neuronal and glial lipid metabolism, and reported mounting evidence that suggests a significant impact of lipid metabolism on neurotransmission.

As part of the central nervous system, the vertebrate retina constitutes a suitable model to investigate the effect of diverse extracellular signals [light, lipopolysaccharide (LPS), oxygen levels, oxidative stress, etc.] under physiological and pathological conditions. A number of papers investigated changes in fatty acid (FA) composition and promotion of oxidative stress might be one of the pathways implicated in retinal degeneration (RD) triggered by continuous LED light exposure of low intensity (Benedetto and Contin). On the contrary, brief pulses of bright blue light cause photic responses in Muller glial cell (MGCs) expressing novel non-visual opsins (Opn3 and Opn5) through intracellular calcium mobilization without affecting cell viability (Rios et al.). This prolonged photosensitivity may play a key role in the retinal physiology presumably regulating cell to cell interaction and glia to neuron communication. In addition, abnormal retina exposure to exogenous LPS administration or high oxygen levels shares a common cellular mechanism of autophagy (Bermúdez et al.; Subirada et al.). Indeed, autophagy mediates retinal pigment epithelium (RPE) cell survival through a pathway modulated by Phospholipase D activity (Bermúdez et al.); whereas, it can also modulate vascular, glial, and neuronal activities in an oxygen-induced retinopathy mouse model (Subirada et al.). Strikingly, the pharmacological regulation of autophagy may offer promising therapeutic strategies to reduce neovascular tufts, persistent gliosis together with the promotion of cell survival in retinal inflammatory and degenerative diseases.

During retina degeneration as in retinitis pigmentosa, photoreceptors may be regenerated by MGCs which acts as stem cells. Demonstrated by Volonté et al., there exists a defective crosstalk between neurons and those MGCs in rd1 retinas that severely impairs the regenerative potential of retinal stem cells. Furthermore, the crosstalk among non-neuronal cells, such as: the RPE cells and retinal neurons, is extensively reviewed by Simón et al. in relation to proliferation, survival, migration, neovascularization, inflammation, and death of retinal cells.

## Lipids and Beyond: New Insights on Function and Dysfunction in the Nervous System

Besides their structural role, lipids have pleiotropic functions in terms of intracellular signaling and metabolism. Insights into lipid metabolism and signaling, lipid-protein interaction, and lipid-binding proteins are topics of many new research papers and reviews. Bioactive lipids inevitably are involved in both physiological and neuropathological processes. FAs have relevant participation as secondary messengers in neuronal signaling. Free FAs are ligands of different types of proteins, such as G-protein coupled receptors, FA-binding proteins, and transcription factors of the Peroxisome Proliferator-Activated Receptors family. The wide variety of signaling molecules modulated by free FAs determine their importance in diverse cellular processes occurring in neurons and glial cells. As new roles for these bioactive compounds are deciphered, new promissory therapeutic targets are under consideration (Falomir-Lockhart et al.). The enzymes catalyzing the elongation of very long chain FAs (ELOV) has been described as new players in neuronal survival and synaptic signaling. ELOV4, one member of this elongase family, expressed in neurons and several mutations in its encoding gene has been associated with different neurological disorders (Stargardt-like macular dystrophy, spinocerebellar ataxia 34, and others) (Deák et al.).

FA-signaling also participates in the sphingolipid rheostat in the retina. Sphingolipids are a complex family of lipids including ceramide, ceramide 1-phosphate, and sphingosine 1-phosphate with relevant roles during development and in the degenerative diseases onsets (Simón et al.). Docosahexaenoic acid, a major n-3 polyunsaturated FA in nervous system, has been shown to protect photoreceptor cells from death through the modulation of the sphingolipid rheostat by decreasing ceramide levels or by enhancing sphingosine 1-phosphate synthesis (Simón et al.).

Phosphatidic acid (PA) is another pleiotropic molecule exhibiting central roles in glycerolipid metabolism and in cellular signaling. Produced by phospholipase D, PA can bind and regulate the activity of an important number of cellular targets such as nucleotide-binding proteins, kinases, and phosphatases. In consequence, deregulation of PA production or catabolism are associated with synaptic dysfunction and several neurological disorder, such as cognitive deficits related to AD, intellectual disability diseases (Fragile-X and Coffin-Lowry syndromes) and fetal alcohol spectrum disorders (Tanguy et al.).

Regarding biological membranes and neurodegenerative diseases such as in AD and PD, a couple of impressive reviews summarizes most of the literature published over the last decades (Alza et al.; Fabiani and Antollini). The article by Fabiani and Antollini highlights the role of different lipids (cholesterol, PA, sphingomyelin, and gangliosides) in nicotinic acetylcholine receptor function, and the crosstalk between amyloid processing, cholinergic signaling and membrane lipid composition, reinforcing the relevance of cholinergic hypothesis for AD. In contrast, lipid-binding properties of α-synuclein, whose pathological aggregation and accumulation are hallmarks of PD has also been described by Alza et al. highlighting the state of the art of how phospholipids, FAs and their metabolisms participate in the pathological aggregation of this protein.

In the context of lipid biology, a transcription factor, c-FOS, may play a critical role. This intriguing protein belongs to the Immediate Early Gene family and apart from its role as transcription factor, has the unique characteristic of regulating *de novo* biosynthesis of lipids and their enzymes by protein-protein interactions at the endoplasmic reticulum. This novel function is essential for membrane biogenesis, cell proliferation and neurite outgrowth, and involved in pathological conditions such as brain tumors growth and development (Rodríguez-Berdini and Caputto).

## Neurodegenerative Diseases: Understanding Normal Physiology Guides Intervention into Pathology

To develop therapies and biomarkers for neurodegenerative diseases, one imperative is to understand the normal physiology of the brain during development and aging. Pro-survival and anti-toxic strategies are approaches to protect neurons and prevent neurodegenerative diseases. In this issue, the use of neurotrophins in neuroprotective strategies is reviewed by Saragovi et al.. Gestational, developmental, and nutritional conditions may have permanent effects on the brain physiology. In this issue, the contributions of Adamo et al. revealed the effects of marginal zinc deficiency (MZD) during gestation. The researchers found major alterations in signal transduction pathways in rats kept on an MDZ diet throughout gestation and beyond (i.e., ERK1/2, *Sox2*, etc.), down-regulation of *Pax6, Tbr2*, and *Tbr1* expression, a lower density of neurons and a selective decrease of glutamatergic neurons in the young adult brain cortex. Collective changes that can potentially result in behavioral impairment throughout life (Adamo et al.).

Estrogens are characterized as signals involved in the sexual differentiation of the brain. Recently, evidence highlighted the participation of estradiol, not only as a reproductive hormone, but also as a brain derived neuronal, protective factor. The coordination of estradiol signaling protects against neurodegenerative diseases and cognitive decline. The work by Zapata et al. elucidates the mechanism of estradiol to promote axonal growth showing that calcium mobilization from the extracellular space and the endoplasmic reticulum is necessary for the ERK1/2 activation and axogenesis in cultures of hypothalamic neurons.

One interesting aspect of signaling mechanisms is that receptor interactions at the neuronal plasma membrane provide a level of regulation. In the report by Soto et al. measurements of neuronal, plasma-membrane receptors co-localization and expression obtained by immunological pull-down experiments in combination with TIRF microscopy and AFM imaging demonstrated that P2X4/5-HT3A receptor complexes can interact with each other in a 1:1 stoichiometric manner, and preserved after ATP binding. Additional information on the receptor binding interaction and the allosteric regulation of its activity is also provided.

Among the neurodegenerative diseases, prion-like protein aggregation is a typical characteristic of each disease. Protein misfolding and signaling abnormalities is correlated and causally related to neuronal pathology and the progression of neurodegeneration. The mechanisms underlying the retrograde neurodegeneration remains elusive. In a review, Zamponi and Pigino highlighted the involvement of fast axonal transport and CK2 activity in the process of neurodegeneration in AD and prion disease. AD, the most common neurodegenerative disease, is diagnosed by two main histopathological lesions: extracellular amyloid plaques mainly composed by the beta-amyloid peptide (Aβ), and the intra-neuronal, neurofibrillary tangles, mainly composed by hyperphosphorylated tau (HP-tau). In this issue, Lasala et al. demonstrated that the interaction of Aβ directly affects α7 nicotinic receptor by acting as an agonist and a negative modulator. Reduced α7 activity, in the presence of higher Aβ concentrations or its long exposure, contribute to the cholinergic signaling deficit, and may be involved in the initiation and development of AD. Moreover, Morozova et al. presented evidence that the muscarinic cholinergic receptor M1/M3 is linked to the tau uptake by the neurons. The uptake of pathological HP-tau induced neurite breakdown. The release and uptake of HP-tau might participate in the prion-like transmission of the disease to neighboring neurons, and preventing this transmission might provide basis for new therapeutic designs.

## Concluding Remarks

In summary, the accumulation of novel research articles and reviews from this topic call support the further expansion of research into cellular and molecular neurobiological processes. Decoding the puzzle of these processes under physiological and pathological conditions can pave the way to identify potential biomarkers and therapeutic targets and novel treatments. A list of these contributions is appended bellow.

## Author Contributions

MG and GS wrote Research Topic proposal. MG, GS, and AA wrote the editorial letter, assigned reviewers, edited articles, and reviewed final manuscript. All authors contributed to the article and approved the submitted version.

## Conflict of Interest

The authors declare that the research was conducted in the absence of any commercial or financial relationships that could be construed as a potential conflict of interest.

